# The Search for Specifics

**Published:** 1898-11-05

**Authors:** 


### THE SEARCH FOR SPECIFICS.

Much sarcasm has been expended upon those who have persisted in hoping that in the good time coming we shall have at our disposal specific cures for definite diseases. It has been considered by many that treatment consists chiefly in placing the patient under conditions which shall be favourable to his recovery; for a long time it has been the fashion to speak as if we were doing all that was possible when we watched our patient through his illness, trusting much to the *vis medicatrix naturæ*, and contenting ourselves with warding off as far as might be such complications as tended towards death, and nothing could be too hard to say of those who confessed to a lingering belief that certain drugs were "good for" certain diseases. Nevertheless, these superior people always had three drugs to reckon with which declined to be deposed from their position as specifics, namely, mercury, iodide of potassium, and quinine. And as time has gone on and knowledge has increased, many things have happened which tend to show, or at any rate to suggest, that, just as in health, certain cells possess a selective power and pick out from the blood stream substances which other cells pass by unnoticed, so in disease there is a selective affinity between various morbid cells and certain substances which, under the circumstances, we can but speak of as specific remedies. The most striking instance of this property which we have before us in the current medicine of to-day is the use of Coley's fluid in the treatment of sarcoma.

Two lectures recently delivered, one by Professor Virchow and the other by Dr. Buzzard, should serve to remind us afresh of the selective affinity which exists between living cells and the constituents of the blood-plasma from which they derive their nutriment. Looking at the matter physiologically, we see each cell leading its own semi-independent life, linked to the rest of the organism by its nervous connection through which it is stimulated to or inhibited from functional activity, but so far as its own vegetative nutrition is concerned feeding upon the pabulum supplied to it, and selecting from the lymph stream such elements as it requires. According to Dr. Buzzard precisely the same thing happens in pathology, and it is the selective affinity of certain cells and groups of cells for the toxins brewed by certain organisms that causes whole tracts of nerve tissue to be affected by disease, while neighbouring tracts remain untouched. We have long been familiar with the same thing in regard to lead and alcohol and some other poisons, and of late years attention has been especially drawn to it in connection with the phenomena of post diphtheritic paralysis. But the same again is true in pharmacology. Not only do we know of direct and specific antidotes, such as the antitoxin of diphtheria, but we see that under the influence of iodide of potassium a solid node dissolves away; under the influence of colchicum an acute and painful inflammation subsides in a night; and under the influence of tuberculin morbid tissues which have been quiescent take on violent reaction; the drug in each case operating by virtue of its special affinity for the morbid cell, or for the toxin which may be present. Coley's fluid, however, carries us still further in the same direction, and introduces us to an entirely new class of specific remedies. This drug consists of the mixed unfiltered toxins of the streptococcus of erysipelas and the bacillus prodigiosus, sterilised by exposure to a moderate heat, and when it is injected, even into parts remote from the tumour, the cells of which the latter consist undergo necrobiotic changes, shrivel, and sometimes slough bodily away. This result may not always follow, but the occurrence of even one positive instance of the absorption of a solid mass like a sarcoma under the influence of an organic toxin injected at a distance opens up an enormous field for speculation and investigation. What is strange, and what is important, is not so much that a drug should be capable of effecting the disappearance of a tumour, but that the drug which has this power should itself be the toxin of another disease.

What is the *modus operandi* of the remedy—whether the toxin acts directly on the morbid cell as is thought by some, or whether it is the disease which it sets up which is the cause of the absorption of the sarcoma, it will not do to speak too positively; but this at least we may say, that the empirical observation that Coley's fluid will cause a sarcoma to melt away is but one more reason for not being too positive that the search for specific remedies for definite diseases is altogether a vain and idle fancy.

